# Treatment of Diphtheria (Local)

**Published:** 1861-02

**Authors:** J. Kneeland


### TREATMENT OF DIPHTHERIA—LOCAL.

BY J. KNEELAND, M. D.

In regard to the local treatment in diphtheria, after consulting physicians who have seen much of the disease in the city of Syracuse and the village of Geddes, and adding their experience to my own, candor compels me to confess, that the so much used and commended "nitrate of silver often fails to remove the false membrane, and change the abnormal action of the secreting surface to a condition of health." In mild cases it is needless, in severe ones too often useless. However, in those terrible sequences of diphtheria, secondary membranous croup, it must be used often, if at all, at least once in three or four hours, and of a strength not less than from forty to sixty grains to the ounce of water, and applied with a curved probang.

To prevent the secretion from forming again after removal (as it often does), I would use tannin and dried alum, applied with a soft linen rag or on the moistened finger to parts within reach, and blown into the throat through an ivory or glass tube, or two or three quills introduced into each other, answer a good purpose; no harm follows inhaling this powder, as a short paroxysm of cough clears all out again.

I apply externally over the throat salt pork, moistened with turpentine, and quickened with pulv. sem. sinapis, or capsicum, if need be. For a gargle, the following is my preference, after trying a variety: R. Hyd. Chlor. Ammoniæ ʒ ij.; Sodæ Muriat. ʒ iv.; Pulv. Capsici ʒ ij.; Vinegar ℥ ij. Add one and a half pints hot water; cool, and use it freely. If any is swallowed, no harm follows. In children too young to gargle, apply with a soft linen rag. In cases where the secretion from the nose, throat, or ear, is offensive, I have used the following: B. Chloride of Soda ʒ ij.; Chlorate of Potash ʒ i.; water, four ounces: with good effects. I tried mur. tinct. ferri as a local application to the throat, and believe it is not as good as nit. argenti.

I have used in a few mild cases, as an internal remedy, a solution of vinegar and muriate of soda in water, covered and quickened by the addition of a little capsicum or piperine, and I must acknowledge that these cases did as well as any that I treated. If I could not get the *chlorine mixture* before described, I should feel able to find in the farmer's pantry, a mixture neither destitute of chlorine nor remedial virtue. The chlorine mixture is usually so well borne by the stomach, so compatible with the use of sustaining food, and, by many years' experience, I have found it so well adapted to the treatment of that congener of diphtheria, scarlatina, that I used in most of my cases from the first; adding a little morphine, or paregoric, if required, and increasing the proportion of acid, or adding the iron if my patient tended downwards, and the stomach would bear this addition.

From what I can gather from the recorded or narrated experience of others, and my own knowledge of the disease as gleaned from a limited number of cases, I have concluded that diphtheria is a blood-changing disease, and that lesion of function in the spinal nerves occurs early in grave cases; that we must not wait for sinking, but if possible anticipate and prevent it; and finally, that to *oxygenate the blood*, and rouse and vitalize the nervous system, are the leading indications to be sought.—*Am. Med. Times.*

